# Supplementary material for: Physical ageing of spreading droplets in a viscous ambient phase
Source: Sci Rep. 2018 Sep 21;8:14159. doi: 10.1038/s41598-018-32392-4 (PMC6155009; doi:10.1038/s41598-018-32392-4)
Supplement: Supplementary file 1 — Supplementary Information [file 41598_2018_32392_MOESM1_ESM.pdf]

Supplementary Information: Physical ageing of spreading droplets in a viscous ambient phase.

Bibin M. Jose,[1] Dhiraj Nandyala,[1] Thomas Cubaud,[1] and Carlos E. Colosquil[1,2*]

[1] Department of Mechanical Engineering, Stony Brook University, Stony Brook, NY 11794, USA

[2] Department of Applied Mathematics & Statistics, Stony Brook University, Stony Brook, NY 11794, USA

[*]carlos.colosqui@stonybrook.edu

1. Derivation of the spreading equation in the near-equilibrium kinetic regime

We begin by considering that sufficiently close to equilibrium the spreading of the studied droplets is the result of random transitions between numerous metastable states (i.e., local minima) in the free energy profile $\mathcal{F}(A)$ [see Equation 2 in the main text], which can be parametrized by the contact area $A = \pi R^2$ where R is the average contact radius of a hemispherical droplet. Hence, the evolution of the contact area can be described by the rate equation

$$\frac{dA}{dt} = A_d(\Gamma_+ - \Gamma_-), \quad \text{(S1)}$$

where A_d is the characteristic base area of topographic surface defects and $\Gamma_\pm$ are the transition rates between the neighboring metastable states in the forward/backward ($\pm$) directions. Adopting Kramers theory of thermally activated escape from a metastable state we have

$$\Gamma_\pm = \frac{\sqrt{\frac{\partial^2 \mathcal{F}_0}{\partial A^2}\left|\frac{\partial^2 \mathcal{F}_\pm}{\partial A^2}\right|}}{2\pi\xi_A} \exp\left(-\frac{\Delta\mathcal{F}_\pm}{k_B T}\right) \quad \text{(S2)}$$

where $\mathcal{F}_0 = \mathcal{F}(A_0)$ and $\mathcal{F}_\pm = \mathcal{F}(A_0 \pm A_d/2)$ are the free energy at the local minimum A_0 and neighboring maxima, respectively, $\Delta\mathcal{F}_\pm = \mathcal{F}_\pm - \mathcal{F}_0$ are the energy barrier in the forward/backward directions, and $k_B T$ is the thermal energy of the system. In addition, Equation S2 involves the damping coefficient ξ_A, which determines the energy dissipation rate $dE/dt = -\xi_A(dA/dt)^2$.

We will further assume that close to thermodynamic equilibrium conditions expected at $A = A_E$ we have $\Delta\mathcal{F}_\pm(A) = \pm K_E A_d(A - A_E)/2 + K_E A_d^2/8 + \Delta\mathcal{F}$ where $K_E = d\mathcal{F}(A_E)/dA$ and $\Delta\mathcal{F}$ is the characteristic energy barrier induced by each surface defect. Employing the damping coefficient at equilibrium $\xi_E = \xi_A(A_E)$ we define the characteristic area spreading rate

$$V_K = \frac{A_d}{2\pi\xi_E}\sqrt{\frac{1}{4}\left(\frac{2\pi}{A_d}\right)^4 \Delta\mathcal{F}^2 - K_E^2} \times \exp\left(\frac{-\Delta\mathcal{F} - K_E A_d^2/8}{k_B T}\right) \quad \text{(S3)}$$

and area $A_K = 2k_B T/K_E A_d$ for the kinetic regime. To proceed with the analysis it is useful to introduce the dimensionless variable $x = (A_E - A)/A_E$, area ratio $\alpha = A_K/A_E$, and damping factor $f(x) = \xi/\xi_E$. In general, the damping factor can be expressed as power series $f(x) = 1 + \sum_{n=1}^{\infty} c_n x^n$ with $f(0) = 1$. For MKT in particular we have $f(x) = (1 - x)^{-1/2}$ from where one can readily determine the coefficients c_n via Taylor expansion. Introducing the dimensionless parameters defined above, the evolution of the dimensionless spreading parameter $x(t)$ is obtained by integrating Equation S1, which gives

$$\int \frac{f(x)}{\sinh\left(\frac{x}{\alpha}\right)} dx = \alpha \log\left[\tanh\left(\frac{x}{2\alpha}\right)\right] + \sum_{n=1}^{\infty} c_n \int \frac{x^n}{\sinh\left(\frac{x}{\alpha}\right)} dx = -\frac{V_K}{A_E} t + c \quad \text{(S4)}$$

where c is an integration constant. The integrals within the series in the left-hand side of Equation S4 for $n \geq 1$ can be expressed as

$$\int \frac{x^n}{\sinh\left(\frac{x}{\alpha}\right)} dx = d_n \frac{x^{n+1}}{n+1} + e_n \alpha^{n+1} + \sum_{m=1}^{n+1} g_{m,n}\, \alpha^m x^{n+1-m} \left[Li_m\left(-e^{-\frac{x}{\alpha}}\right) - Li_m\left(e^{\frac{x}{\alpha}}\right)\right], \tag{S5}$$

where Li_m is the m-order polylogarithm function and d_n, e_n, and $g_{m,n}$ are real numbers whose values are known after analytical integration. In particular, we have $d_n = e_n = 0$ for $n = 1,2$, $e_n = -1$ for $n \geq 3$, and $g_{1,n} = 1$ for $n \geq 1$. Further considering that $Li_1\left(-e^{-\frac{x}{\alpha}}\right) - Li_1\left(e^{\frac{x}{\alpha}}\right) = \log\left[\tanh\left(\frac{x}{2\alpha}\right)\right]$ and introducing Equation S5 into Equation S4 we arrive to

$$f(x) \log\left[\tanh\left(\frac{x}{2\alpha}\right)\right] + O(\alpha^2) = -t/T_K + c, \tag{S6}$$

where $T_K = \alpha A_E/V_K = A_K/V_K$ is the kinetic time expressed in Equation 7 in the main text. Hence, Equation S6 gives an approximate implicit relation for the evolution of the contact area $A(t) = A_E(1-x)$. For the case that the equilibrium contact area $A_E \gg A_K$ is much larger than the "kinetic" area and thus $\alpha \ll 1$, Equation S6 is equivalent to Equation 6 in the main text, given that $x/\alpha \gg 1$ for $t = 0$ and thus the integration constant is $c \simeq 0$. Furthermore, for $x \ll 1$ we find that Equation S6 can be approximated by the explicit expression $x = 2\alpha\, \text{atanh}[\exp(-t/T_K)]$. Considering that $t/T_K < 1$, the latter expression for x predicts a logarithmic-in-time relaxation for the contact area

$$A(t) = A_E + A_K \log\left(\frac{t}{2T_K}\right). \tag{S7}$$

It is worth noticing that Equation S7 is the same expression as Equation 8 in the main text, which is given in terms of the contact radius $R(t)$. Equation S7 indicates that the contact area in the near-equilibrium spreading regime follows a nearly logarithmic evolution in time.

2. Determination of damping coefficients from experimental data

The damping coefficients employed in analytical expressions for the near-equilibrium spreading regime have been determined from our experimental observations in the initial spreading regime where there are no local minima in the free energy profile $\mathcal{F}(A)$ and therefore no metastable states are present. Under such conditions, Equations 1 to 3 in the main text give the dynamic equation $\xi \times dR/dt = -2\pi R\gamma(\cos\theta - \cos\theta_E)$ for the average contact radius $R(t) = \sqrt{A/\pi}$ assuming a spherical droplet. In the dynamic equation for R, γ is the water-oil surface tension, θ and θ_E are the non-equilibrium and expected equilibrium contact angle, respectively, and $\xi = \xi_A \times 2\pi R^2$ is the conventional damping coefficient for contact line displacement that can be predicted via the Molecular Kinetic Theory (MKT), as discussed in the main text. From experimental observations for the contact radius and its displacement rate we therefore evaluate the damping coefficient

$$\xi(R) = -\frac{2\pi R\gamma(\cos\theta - \cos\theta_E)}{\frac{dR}{dt}}, \tag{S8}$$

which involves assuming that the droplet is a hemispherical cap and therefore the non-equilibrium contact angle $\theta = \theta(R; V)$ is determined from the contact radius for a given droplet volume. The equilibrium contact angle employed in Equation S8 is $\theta_E = 75°$, which corresponds to the mean value observed for both droplets in mechanical equilibrium and in Wilhelmy plate measurements.

Results from Equation S8 are reported in Figure S1 for droplets with different volume $V = 0.24$ to $0.64\ \mu$L. Notably, we observe a nearly linear relation between the damping coefficient $\xi \propto R$ when the contact radius $R < R_C$ is smaller than the crossover radius R_C predicted by Equation 9 in the main text (see vertical dashed lines in

Figure S1). Hence, the damping coefficient determined by experimental observations in the initial dynamic regime where $R < R_C$ can be rationalized by MKT according to which according to which $\xi = \chi\mu_o 2\pi R$ and χ =110 to 175 (see Figure S1) is a friction factor determined by physicochemical properties of the liquid and solid. It is worth noticing that for the studied liquid-liquid system the viscosity μ_o = 96 mPa of the ambient phase (100 cSt silicone oil) is about 100 times larger than the viscosity of the droplet phase (DI water) and thus analytical estimations consider only contributions to damping from the ambient phase.

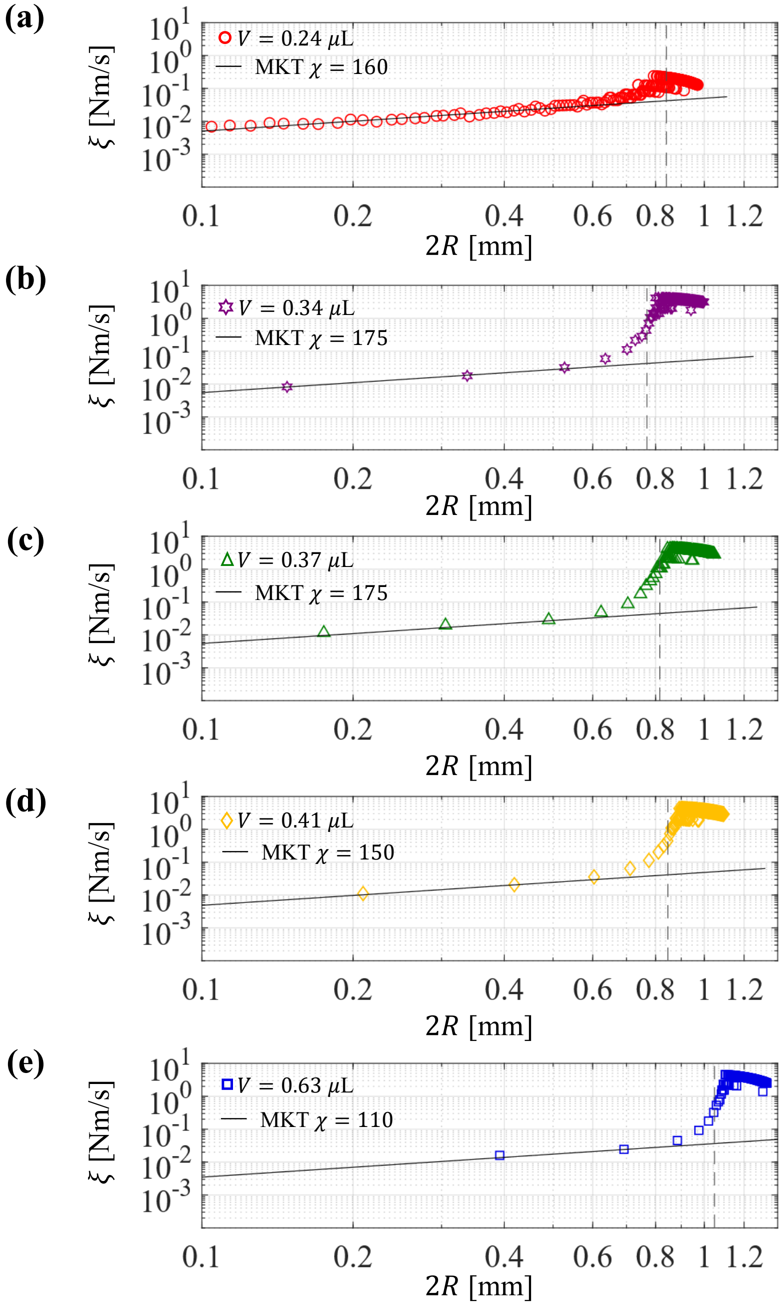

Figure S1: Damping coefficient ξ versus contact radius R for droplets of different volume. (a) $V = 0.24$ μL. (b) $V = 0.34$ μL. (c) $V = 0.37$ μL. (d) $V = 0.41$ μL. (e) $V = 0.63$ μL. Markers: experimental results obtained by Equation S8 for $\theta_E = 75°$. Solid lines: MKT predictions, friction factors χ are determined by fitting the experimental results. Dashed vertical lines: crossover radius R_C predicted by Equation 9 in the main text for $\alpha = 0.55$.

Based on analysis of the experimental results reported in Figure S1 we adopt MKT predictions with friction factors χ obtained by empirical fitting in order to determine the damping coefficients $\xi_E = \chi\mu 2\pi R_E$ employed in analytical expressions for the near-equilibrium kinetic regime. It is worth noticing that the effective damping coefficient determined by Equation S8 sharply increases nearly two orders of magnitude around the predicted crossover radius $R \simeq R_C$ beyond which the observed slow spreading process, or physical ageing, is attributed to thermally activated transitions between metastable states in the modeled free energy profile (see Equations 1-2 in the main text). Our experimental findings thus indicate that MKT predictions can account for the spreading dynamics observed for $R < R_C$. However, the near equilibrium spreading behavior observed for $R > R_C$ cannot be accounted for by the damping coefficients $\xi \propto R$ predicted by MKT. Therefore, we conclude that solely considering damping effects due to dissipation of energy associated with the adsorption/desorption of liquid molecules at the contact line is not sufficient to describe the slow near-equilibrium spreading regime observed in our experiments.
